# Multiple copy number variants of *VC1* gene reveal single-copy expression as a key determinant of vicine content

**DOI:** 10.3389/fpls.2025.1565210

**Published:** 2025-06-13

**Authors:** Samson Ugwuanyi, Manar Makhoul, Agnieszka A. Golicz, Christian Obermeier, Rod J. Snowdon

**Affiliations:** Department of Plant Breeding, Justus Liebig University, Giessen, Germany

**Keywords:** VC1 gene, structural variation, KASP markers, *Vicia faba*, convicine

## Abstract

Faba bean is a valuable legume crop desired globally for its high nutritional composition. However, the seed vicine and convicine (v-c) content reduces the nutritional quality of faba bean protein and can induce favism in susceptible individuals. Recently, *VC1* gene, encoding a bi-functional riboflavin protein, was reported to initiate the v-c biosynthetic pathway in *V. faba*. In low v-c cultivars, a 2 bp insertion in this gene disrupts its function by causing a frameshift and premature stop codon. However, because v-c biosynthesis is only partially reduced, this suggests that additional genes may also be involved in the pathway. Here, we identify and investigate multiple tandem gene duplications at the *VC1* locus. Our findings reveal that *VC1* exhibits multiple structural variants and copy number variations, but its expression is independent of copy number. Low v-c genotypes carry both variants of the gene — with and without the 2 bp insertion — but only the variant with the insertion is expressed. In contrast, high v-c genotypes consistently express the variant lacking the insertion. Although some high v-c genotypes also carry the insertion, it is found in a non-expressed variant, while the expressed variant lacks the insertion, resulting in the high v-c phenotype. We also report a novel diverging *VC1* homolog, *RIBA2*, which shares expression domains with *VC1*. This homologous gene encodes GTP cyclohydrolase II, a critical enzyme in the v-c pathway. Expression of this gene contributes ~5-10% of riboflavin gene transcripts in immature seeds suggesting it as a minor-effect candidate locus in v-c biosynthesis. Moreover, two SNPs within the coding sequence of *RIBA2* segregated with v-c content, offering a reliable alternative for marker-assisted selection in faba bean breeding. In conclusion, this study contributes to the elucidation of the complex genetic regulation of v-c biosynthesis and provides valuable insights to facilitate further efforts in its reduction in faba bean.

## 1 Introduction

Faba bean (*Vicia faba* L.) is a grain legume which is globally important for its highly nutritious, protein-rich seeds. The global production of faba bean stands at 5.7 million tons in 2020 (FAOSTAT, 2022), making it the sixth most produced pulse crop and the highest yielding legume after soybean (Adhikari et al., 2021). It is reported to have originated from the Mediterranean basin and subsequently spread to be cultivated across nearly all continents worldwide (Duc et al., 2010; Caracuta et al., 2016). It has become increasingly popular, especially in cool-season climates where other protein crops perform poorly. Faba bean seeds contain up to 37% protein and are rich in micronutrients, making them a suitable source of food for humans and feed for their livestock (Duc et al., 1999; Karkanis et al., 2018; Warsame et al., 2020). In addition to its nutritional benefits, faba bean can improve soil fertility in association with rhizobium bacteria by fixing a significant quantity of nitrogen in the soil, which reduces the need for application of inorganic nitrogen fertilizer in subsequent seasons (Karkanis et al., 2018; Adhikari et al., 2021). This feature is leveraged in many agricultural systems by incorporating faba bean into crop rotation or mixed cropping with others crops such as cereals (Stagnari et al., 2017; Carrillo-Perdomo et al., 2020). This ecological importance of faba bean and its roles in food and feed has increased its global reputation significantly.

However, the agronomic relevance of faba bean is limited by the presence of significant quantities of vicine and convicine (v-c) in all parts of the plants (Luzzatto and Arese, 2018; Choudhary and Mishra, 2019; Badjona et al., 2023). The metabolic products of vicine and convicine—divicine and isouramil—release free radicals that cause oxidative damage to red blood cells in people with glucose-6-phosphate dehydrogenase (G6PD) deficiency, leading to acute hemolytic anemia, a condition also known as favism (Badjona et al., 2023; Björnsdotter et al., 2021). Therefore, to enhance faba bean usage and its general acceptability, reducing the v-c content to the barest minimum safe for food and feed is essential. However, the genetic basis of v-c accumulation remains to be fully elucidated, and all low v-c cultivars still carry baseline v-c levels.

Previous studies have highlighted a bimodal pattern in the v-c phenotype, primarily influenced by a major quantitative trait locus (QTL) on chromosome 1 of the faba bean genome (Duc et al., 1989; Ramsay et al., 1995; Gutierrez et al., 2006; Khazaei et al., 2015). Molecular markers flanking this locus were identified by Khazaei et al. (2015); however, the gene *RIBA1* was only recently discovered within this region by Björnsdotter et al. (2021). *RIBA1*, named *VC1*, encodes a bi-functional riboflavin protein responsible for catalyzing the pivotal step in the v-c biosynthetic pathway. The gene has two functional domains, RibA and RibB, encoding GTP cyclohydrolase II (GCHII) and 3,4-dihydroxy-2-butanone-4-phosphate synthase (DHBPS), respectively. However, it is the GTP cyclohydrolase II domain that directly catalyzes v-c biosynthesis, via conversion of purine nucleoside triphosphate GTP into the unstable intermediates leading to v-c (Björnsdotter et al., 2021). A 2 bp insertion in the GCHII domain leads to loss of function in low v-c cultivars. This frameshift mutation inactivates *VC1* by causing a premature stop codon, hindering the correct synthesis of GTP cyclohydrolase II. However, this mutation does not eliminate v-c completely, although it causes a significant reduction. This suggests a potential involvement of other genes or gene copies, necessitating further research to comprehensively understand the genetic factors that control v-c biosynthesis in faba bean. Recently completed *V. faba* genome assemblies and gene annotations for low and high v-c cultivars (Jayakodi et al., 2023) provide an excellent basis to advance knowledge in this regard. Therefore, the objectives of this study were to elucidate the genetic regulation of v-c content by identifying active bi-functional riboflavin genes and polymorphisms corresponding to changes in v-c content. Due to the residual v-c content in seeds of low v-c genotypes, we hypothesize that at least one additional locus controls v-c biosynthesis. To study this, we identified RIBA protein-coding genes by homology mapping of the RIBA1 protein sequence to two faba bean reference genomes with high and low v-c content, respectively. All similar protein-encoding genes were identified and functionally analyzed across a set of well-characterized low and high v-c cultivars using bioinformatic predictions, gene expression and transcript analyses, and phenotype correlation analysis.

## 2 Materials and methods

### 2.1 Plant materials and cultivation

The study utilized a diverse set of well-characterized faba bean lines consisting of nine low v-c and nine high v-c genotypes with different genetic backgrounds (**Supplementary Table S1**). These lines represent a set of carefully selected genotypes with known characteristics and performances. They were obtained from Norddeutsche Pflanzenzucht Hans-Georg Lembke KG (NPZ, Hohenlieth, Germany) and comprise both commercial cultivars and lines inbred beyond the fifth generation of selfing. Vicine content for each genotype was measured from a sample of two faba bean seeds using spectrophotometry as described by Sixdenier et al. (1996).

Two seeds from each genotype were planted in 4-liter plastic pots within a pollinator-proof chamber in the greenhouse. Fresh leaves at 21 days after planting were collected from the first fully opened leaves for DNA isolation. For RNA extraction for gene expression and transcript analyses, a subset of ten genotypes was selected, including five with low and five with high seed v-c content. The plants were manually tripped during flowering to ensure pod-set (Kambal et al., 1976). Fresh and immature seeds were collected from this subset for RNA isolation at two stages during seed development: early seed filling stage (ESF) (stage four) and late seed filling stage (LSF) (stage six). Specifically, seeds aged 13–16 days and 20–25 days after tripping were selected, respectively. At the ESF stage, the cleft between the cotyledons is broad, with a spherical chalazal chamber. The embryo has a butterfly-shaped appearance and is surrounded by endosperm. In contrast, at LSF, the intense green cotyledons are closely positioned and have a curved axis (Borisjuk et al., 1995).

### 2.2 DNA extraction

Fresh leaves collected from each genotype were immediately flash-frozen in liquid nitrogen and lysed using Qiagen Tissue Lyser II (Qiagen, Düsseldorf, Germany). Genomic DNA was isolated from the leaf powder following the Doyle and Doyle (1990) method.

### 2.3 RNA extraction and cDNA synthesis

Immature seeds were immediately flash-frozen in liquid nitrogen and then manually ground into powder using a mortar and pestle. RNA was isolated from 100 mg of the powdered samples using the Zymo RNA miniprep kit (Zymo Research, Freiburg, Germany) following the manufacturer’s manual. DNaseI treatment was performed to remove genomic DNA, following the procedure outlined in the Zymo RNA kit manual. RNA concentration and quality were determined using Qubit RNA assay kit (ThermoFisher Scientific, Germany) and agarose gel (1%), respectively. First-strand cDNA was synthesized using the RevertAid cDNA synthesis kit (ThermoFisher Scientific, Germany). Initially, 1 µl of Random Hexamer primer was added to 1 µg of RNA, incubated for 5 minutes at 65°C, and subsequently cooled on ice. The cDNA reaction master mix was prepared by adding 4 µl of Reaction buffer (5x), 2 µl of dNTP mix (10mM), 1 µl of Ribolock RNase inhibitor (20 U/µl), and 1 µl of RevertAid H Minus Reverse Transcriptase (200 U/µl). Reactions were carried out in a thermal cycler at the following temperature conditions: 25°C for 5 minutes, 42°C for 60 minutes, 70°C for 5 minutes, and then held at 4°C. The resulting cDNA was utilized for gene expression analysis and Sanger sequencing of *VC1* and *RIBA2* genes.

### 2.4 Homology-based gene identification

Custom databases for faba bean were established from the reference assemblies of the high v-c cultivar, Hedin, and the low v-c cultivar, Tiffany (Jayakodi et al., 2023), using ncbi-blast 2.12.0+. RIBA1 protein sequence was aligned to these databases using the tblastn function to identify all RIBA genes in both genomes. Genes exhibiting sequence similarity were identified and filtered to retain only those with over 85% protein sequence similarity to RIBA1, and were then functionally analyzed in this study.

### 2.5 Phylogenetic and sequence analysis

Alignments were performed using MAFFT tool in Jalview (version 2.11.3.0). Phylogenetic analysis was conducted using the phylogeny.fr platform. The maximum likelihood method, implemented in the PhyML program (v3.1/3.0 aLRT), was employed to reconstruct the phylogenetic tree while TreeDyn (v198.3) was used for tree rendering. Amino acid sequences of RIBA proteins for chickpea (XP_004485599), lupin (KAE9591829), grass pea (CAK6822722), lotus (XP_057429064), Medicago (XP_003593237) and pea (XP_050880829) were downloaded from NCBI database (https://www.ncbi.nlm.nih.gov/). Functional domains of RIBA proteins were predicted using PsiPred workbench (http://bioinf.cs.ucl.ac.uk/psipred/).

### 2.6 Primer design and synthesis

All primers for the experiments were designed using Primer3 plus (https://www.primer3plus.com/index.html) and subsequently synthesized by Microsynth AG (Balgach, Switzerland). For each primer, gene sequences from the two reference genomes, Hedin and Tiffany (Jayakodi et al., 2023), were aligned to identify conserved regions with priming efficiency, as predicted by Primer3 Plus.

### 2.7 PCR Validation of *VC1* and *RIBA2* genes in faba bean

All *VC1* variants and *RIBA2* were validated in faba bean genotypes using selective PCR amplification. PCR reactions were set up in a final volume of 25 µl, including 12.5 µl of GoTaq Hot Start Green Master Mix, (Promega, Madison, WI, United States), 1.25 µl of 10 µM forward and reverse primers (**Supplementary Table S2**), 1.5 µl of genomic DNA and 8.5 µl of MilliQ water. The reactions were carried out in a T100 Thermal Cycler (Bio-Rad Laboratories, Hercules, CA, United States) with the following conditions: 94°C for 2 minutes, 35 cycles of denaturation at 94°C for 30 seconds, annealing at 60°C for 30 seconds and extension at 72°C for 40 to 90 seconds depending on the size of amplicon, followed by final extension for 5 minutes at 72°C. Amplicons were separated on a 1% agarose gel and visualized under UV light.

### 2.8 *VC1* copy number determination by quantitative PCR

*VC1* copy number was determined by quantitative PCR in 10 µl final volume, containing 5 µl 2x SYBR Green master mix (ThermoFisher Scientific, Germany), 1 µl 10 µM forward primer and 1 µl 10 µM reverse primer (**Supplementary Table S2**), 1 µl of DNA and 2 µl of MilliQ water. ELF1A was used as the reference gene for normalization (Gutierrez et al., 2011). There were two biological and three technical replicates for each genotype, as well as triplicates of water samples serving as no-template controls. Quantitative PCR was done using StepOneplus (ThermoFisher Scientific, Germany) with the following temperature conditions: 95°C for 10 minutes, 40 cycles of denaturation at 95°C for 15 seconds followed by 60°C for 1 minute. Relative quantification was determined by delta-delta CT (ΔΔCt) method (Livak and Schmittgen, 2001).

### 2.9 Relative quantification of *VC1* and *RIBA2* expression levels by reverse-transcription quantitative PCR

The expression levels of *VC1* and *RIBA2* were determined from cDNA synthesized from the previous step. The reaction mixture consisted of 5 µl of 2x SYBR Green master mix (ThermoFisher Scientific, Germany), 1 µl of 10 µM forward primer and 1 µl of 10 µM reverse primer (**Supplementary Table S2**), 1 µl of cDNA, and 2 µl of MilliQ water. The reference genes, CYP2 and ELF1A, were used for normalization as they were previously reported to be stably expressed in faba bean (Gutierrez et al., 2011). As controls, three water samples were included as no-template controls and each sample had two biological and three technical replicates. Quantitative PCR was done using StepOneplus (ThermoFisher Scientific, Germany) as described above for copy number quantification and relative transcript levels were determined by delta-delta CT (ΔΔCt) method (Livak and Schmittgen, 2001).

### 2.10 *VC1* and *RIBA2* cDNA sequencing

The cDNA samples, synthesized as described above, were used for selective amplification of *VC1* and *RIBA2*. Forward and reverse primer pairs specific to *VC1* and *RIBA2* were used (**Supplementary Table S2**). The PCR conditions were as described previously in the gene validation section, except that the annealing temperature was set at 58°C. Subsequently, the resulting amplicons were sent for Sanger sequencing at Microsynth AG (Balgach, Switzerland). For each genotype, samples from the two developmental stages were sequenced.

### 2.11 KASP marker assay development and SNP genotyping

Genotyping was performed using Kompetitive Allele-Specific PCR (KASP) assay technique for polymorphisms within *VC1* gene. *VC1* genes from the two reference assemblies were aligned to identify possible single nucleotide polymorphisms. For identified variants, allele-specific primers were designed. To detect the presence of 2 bp insertion in *VC1* (SNP08) within our faba bean set, primers were designed where A1 binds to the wild-type variant, and A2 binds to the mutant variant. The common primer used was C1. Detailed information on all *KASP* markers can be found in **Supplementary Table S3**. The KASP marker assay procedure was conducted according to the methodology outlined in the study by Makhoul and Obermeier (2022).

### 2.12 Data analysis

All experiments, including PCR, sequencing, copy number quantification, gene expression analysis, and KASP genotyping, were repeated at least twice. The resulting qPCR data from copy number quantification and gene expressions were analyzed using Microsoft Excel and R (version 4.3.2). The library packages ggpubr and ggplot2 were used to generate plots using R studio. Statistical differences were inferred using t-test for two groups or one-way ANOVA for more than two groups.

## 3 Results

### 3.1 Multiple gene models encode RIBA proteins in the *Vicia faba* genome

**Figure 1:**
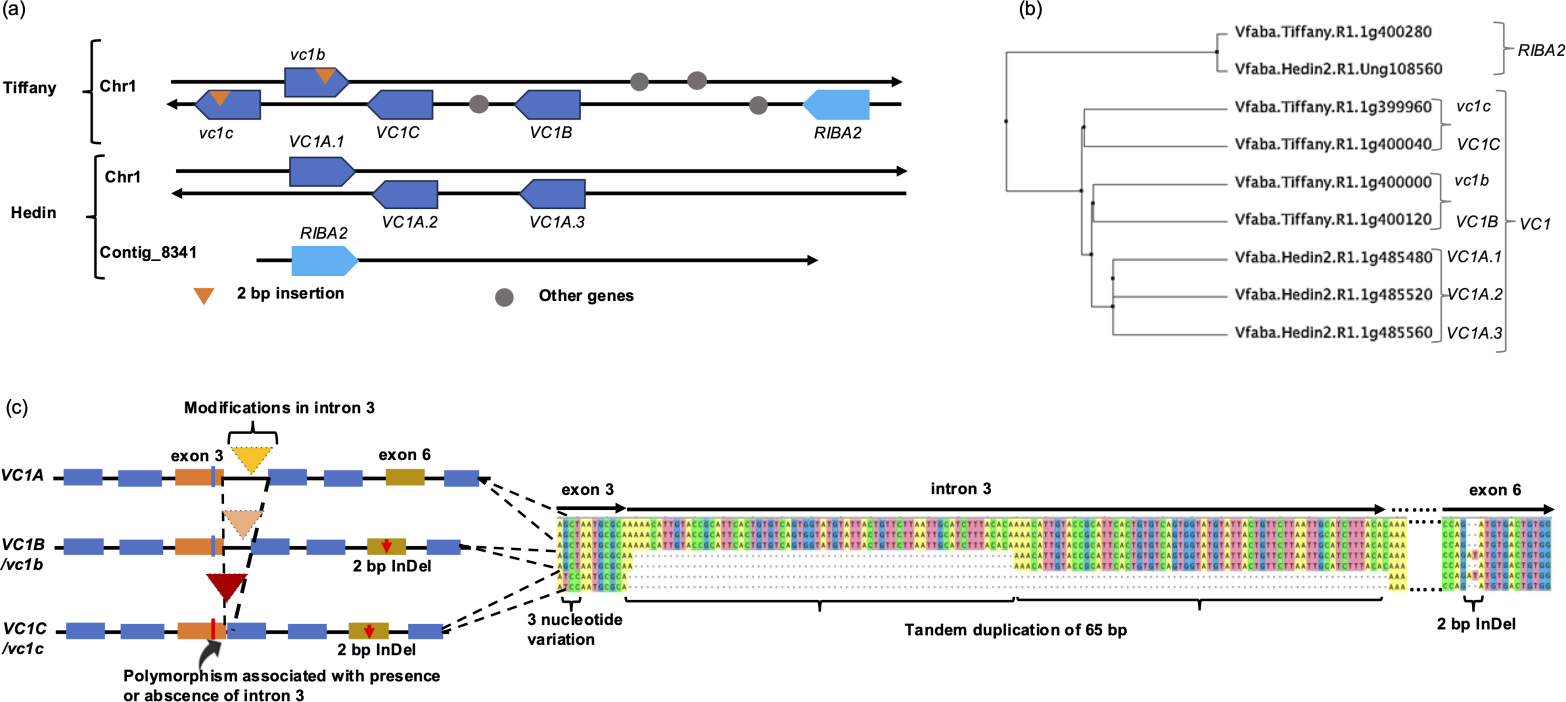
Identification and analysis of RIBA genes. **(a)** Configurations of RIBA genes from two reference assemblies. The direction of arrows shows the orientation of the genes on the top and bottom strand of chromosome 1 and Contig_8341. **(b)** Phylogenetic tree showing the genetic relationship among RIBA genes in *V. faba*. **(c)** Major structural variations in *VC1* genes. *VC1* variants were structurally differentiated by modifications in intron 3. These modifications are associated with a 3-nucleotide variation in exon 3.

In this study, we investigated the role of the different genes that encode bifunctional RIBA enzymes responsible for catalyzing the initial step in the v-c biosynthetic pathway. We employed a homology-based method to align RIBA1 protein to the recently assembled faba bean reference genomes, Hedin— a high v-c cultivar— and Tiffany— a low v-c cultivar (Jayakodi et al., 2023). Our analysis revealed the presence of multiple gene models encoding RIBA protein in the faba bean genome. In Hedin, we identified four such genes on chromosome 1 and contig_8341, namely Vfaba.Hedin2.R1.1g485480, Vfaba.Hedin2.R1.1g485520, Vfaba.Hedin2.R1.1g485560, and Vfaba.Hedin2.R1.Ung108560. Tiffany, on the other hand, had five genes on chromosome 1, including Vfaba.Tiffany.R1.1g399960, Vfaba.Tiffany.R1.1g400000, Vfaba.Tiffany.R1.1g400040, Vfaba.Tiffany.R1.1g400120, and Vfaba.Tiffany.R1.1g400280 (**Figure 1a**; **Supplementary Table S4**). Comparative analysis indicated that three of these genes from Hedin and four from Tiffany shared significant identity (>96%) with the *RIBA1* (*VC1)* gene previously reported by Björnsdotter et al. (2021) (**Figure 1b**). Therefore, we classified these genes as genetic variants of *VC1*. These *VC1* variants exhibited contrasting structural variations that grouped into three major variants, defined by their structural characteristics. *VC1A* had a tandem duplication of a 65 bp in intron 3 and *VC1B* had a partial deletion in this intron, while *VC1C* lacked the entire intron (**Figure 1c**). There were three copies of *VC1A* in the Hedin assembly and two copies each of *VC1B* and *VC1C* in Tiffany. As expected, one copy each of *VC1B* and *VC1C* carried a 2 bp frameshift insertion in exon 6, as reported by Björnsdotter et al. (2021), which renders the gene product non-functional. This insertion was absent in *VC1A*. The modification in intron 3 is associated with a three-nucleotide difference in exon 3 and can distinguish *VC1C/vc1c* from *VC1A* and *VC1B/vc1b*. Beside these variations, *vc1b* and *VC1C* share similar alleles at most SNP positions. Additionally, there are SNPs within exon 4 and 5 that can distinguish other *VC1* variants (**Supplementary Figure S1**).

The fourth and fifth gene models identified in Hedin and Tiffany, respectively, shared only approximately 65% sequence similarity with *VC1* and 99% sequence similarity with each other, suggesting that they represent a newly discovered *VC1*-like gene. While there were structural variations between *VC1* and this homolog, these differences occurred primarily in non-coding regions, with the coding regions displaying a high degree of similarity (>87%). As a result, they shared over 90% similarity in their predicted protein sequences.

**Figure 2:**
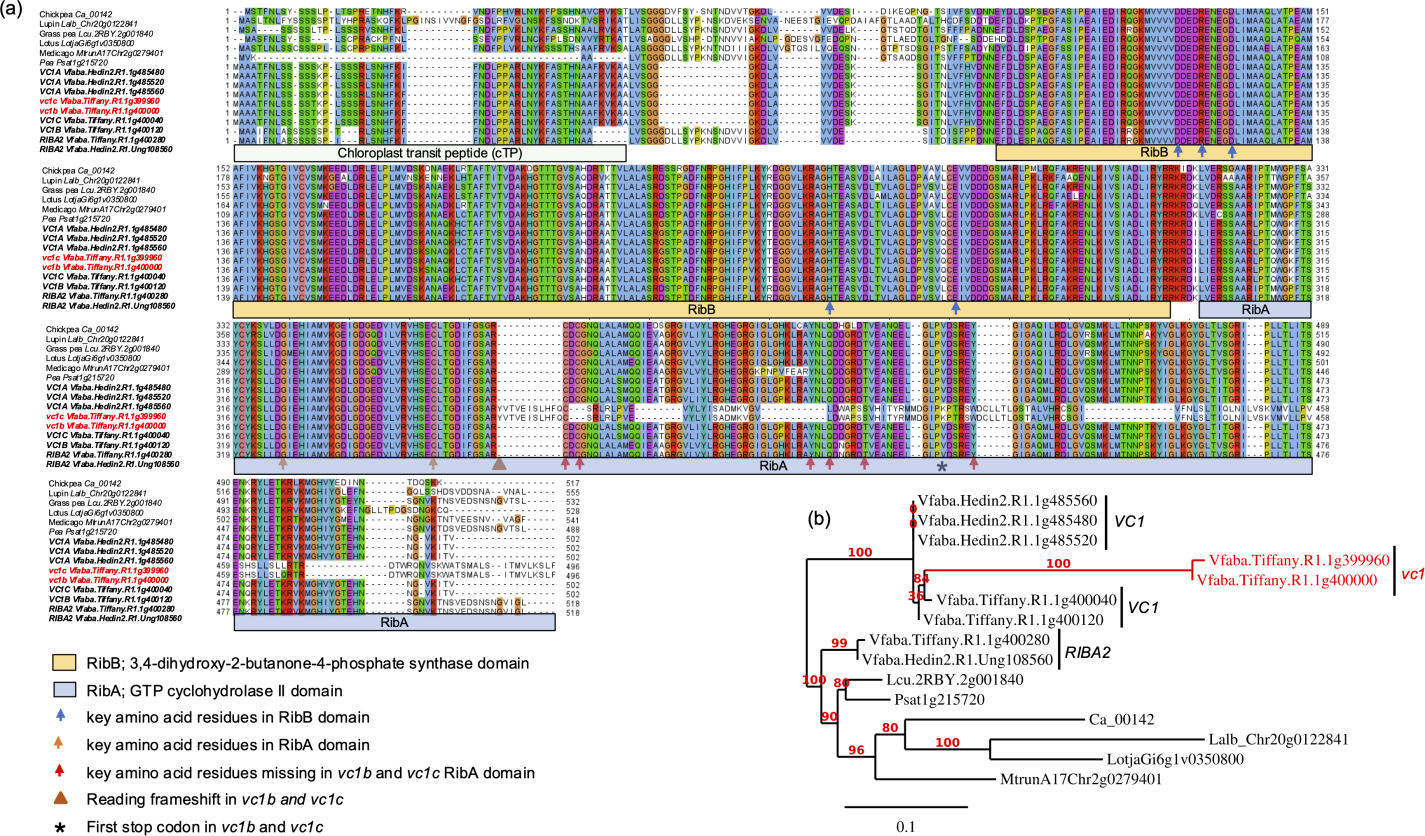
Comparison of amino acid sequences and phylogeny of RIBA proteins. **(a)** Alignment of amino acid sequences and **(b)** phylogenetic tree of RIBA proteins from *V. faba* and related legume crops in Fabaceae family, including chickpea, lupin, grass pea, lotus, Medicago and pea. Major variations lie within the domain encoding chloroplast transit peptide (cTP). RibB and RibA domains of the proteins show high similarity, except the protein sequences encoded by *vc1b* and *vc1c* which show variations in the RibA domain due to a frameshift mutation. The functional domains are shown underneath the alignment as predicted using PsiPred workbench. Predicted key residues based on Björnsdotter et al. (2021) are indicated with different colors of arrows.

Analysis of the protein sequence of this homologous gene showed presence of two functional domains, RibA and RibB, as in other bifunctional riboflavin proteins, and highly identical to *VC1* protein domains. Subsequently, we analyzed bifunctional RIBA protein homologs from other legume crops in the Fabaceae family including chickpea, lupin, grass pea, lotus, Medicago and pea. Comparison of the amino acid sequence encoded by this newly identified *V. faba* gene to those of *VC1* and RIBA genes from other legumes revealed high similarity among these RIBA proteins and conservation of all key amino acid residues required for catalytic activity. All the proteins shared a high identity in the two catalytic domains, except *vc1b* and *vc1c* which differed significantly in the second domain due to a 2 bp insertion, altering the reading frame and causing a premature stop codon (**Figures 2a, b**). Hence, the high degree of similarity among these proteins, especially within their functional domains, suggests that their roles as RIBA proteins are well conserved. We therefore denominated this novel homolog as *RIBA2*, since it represents a second RIBA locus in *V. faba*.

**Figure 3:**
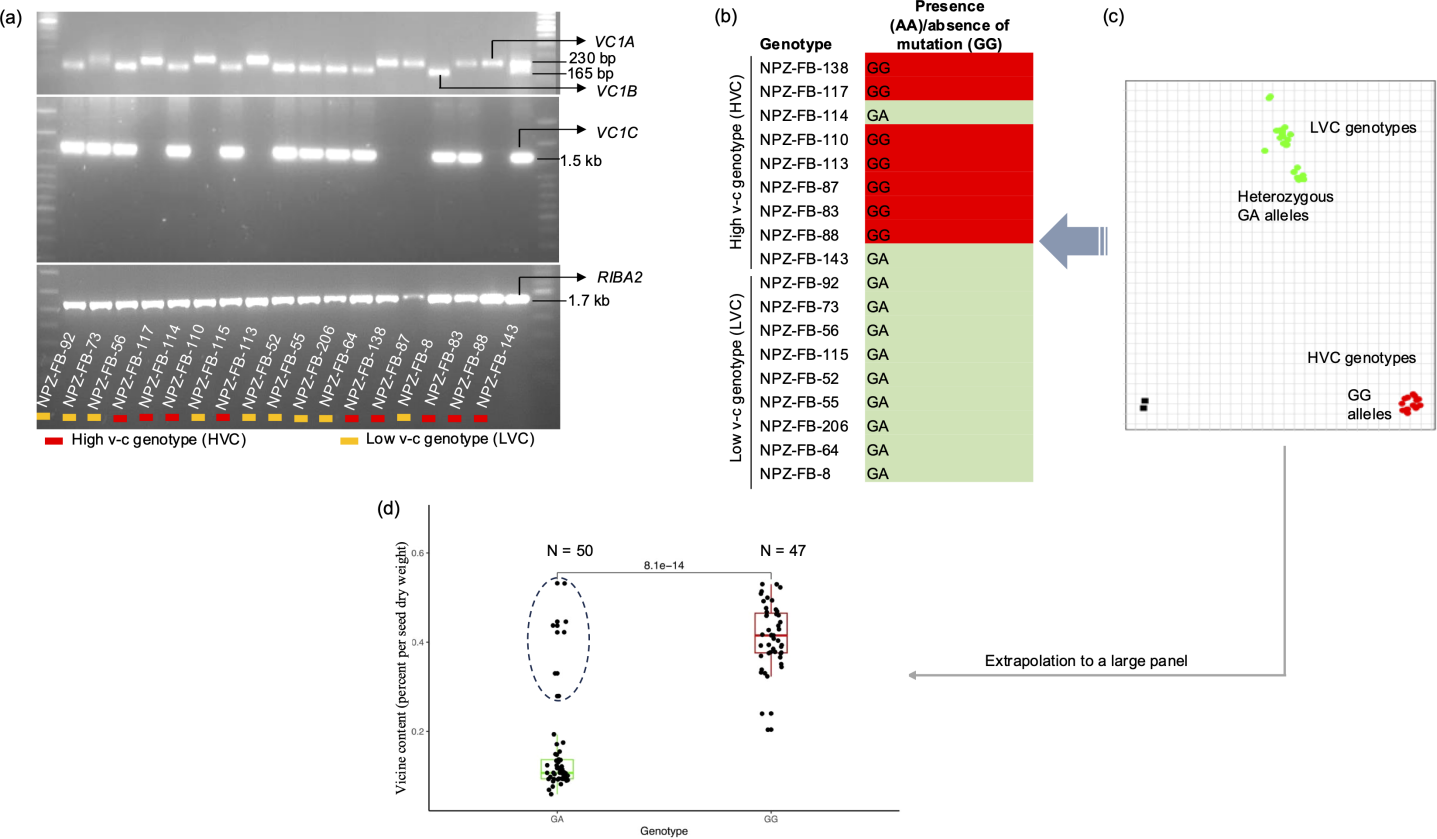
Validation of the presence of RIBA genes and the mutation in *VC1*. **(a)** Selective amplification by PCR showing the presence of *VC1* and *RIBA2* variants in a set of 18 faba bean lines. **(b)** Allele calls and **(c)** allelic discrimination plot for the 2 bp insertion in exon 6 of *VC1*. Allele GG represents the absence of insertion (wild type variants), while allele AA represents the presence of insertion (mutant). Heterozygous cluster (GA) indicates the presence of both the wild type and mutant variants. **(d)** Boxplot illustrating the v-c contents for two groups: *VC1* wildtypes (GG) and genotypes carrying both the mutant and wildtype (GA) gene variants in a panel of 97 genotypes. High v-c genotypes carrying GA alleles are enclosed within a circle.

We validated the existence of all the genes in a set of 18 faba bean genotypes. First, we selectively amplified the genes using PCR. The results indicated that *RIBA2* was present in all faba bean genotypes. *VC1A* was present in most high v-c genotypes, while *VC1B* and *VC1C* were found in all low v-c genotypes but were also carried by some high v-c genotypes (**Figure 3a**). It was also observed that all three *VC1* variants could be present in a single genotype, as observed in the low v-c genotype, NPZ-FB-73, and the high v-c genotype, NP-FB-143. Secondly, the presence of active and inactive copies of *VC1B* and *VC1C* in the Tiffany genome assembly suggests that low v-c genotypes may still carry functional copies of *VC1*. To confirm the hypothesis that both active and inactive copies of *VC1* are carried by low v-c genotypes, we employed a KASP assay targeting the 2 bp insertion in exon 6. KASP primers were designed such that allele 1-specific primers bind to the active variants while allele 2-specific primers bind to the mutated variants. Subsequently, it was observed that most high v-c genotypes (7 out of 9) were homozygous for the assay indicating the presence of only active variants, whereas all nine genotypes with low v-c and two high v-c genotypes were heterozygous, indicating the presence of both active and mutated variants (**Figures 3b, c**). There was no homozygous genotype call for only the mutant, revealing that no faba bean genotype carried only the mutant gene copies. However, since a set of 18 faba bean genotypes is too small to draw definitive conclusions, we extended our analysis to a larger and more diverse panel of 97 genotypes to test this hypothesis. This diversity panel included breeding lines and cultivars exhibiting a wide range of seed v-c content, obtained from Norddeutsche Pflanzenzucht Hans-Georg Lembke KG (NPZ, Hohenlieth, Germany). We observed that all low v-c genotypes, as well as some high v-c genotypes, carried a functional *VC1* variant in addition to the non-functional *vc1* allele (**Figure 3d**; **Supplementary Table S5**), confirming the hypothesis that all low v-c faba beans carry multiple *VC1* variants, including gene variants with and without the inactivating insertion. Additionally, some high v-c faba beans exhibit the same characteristic.

### 3.2 *VC1* exhibits copy number variations and is dosage-insensitive

**Figure 4:**
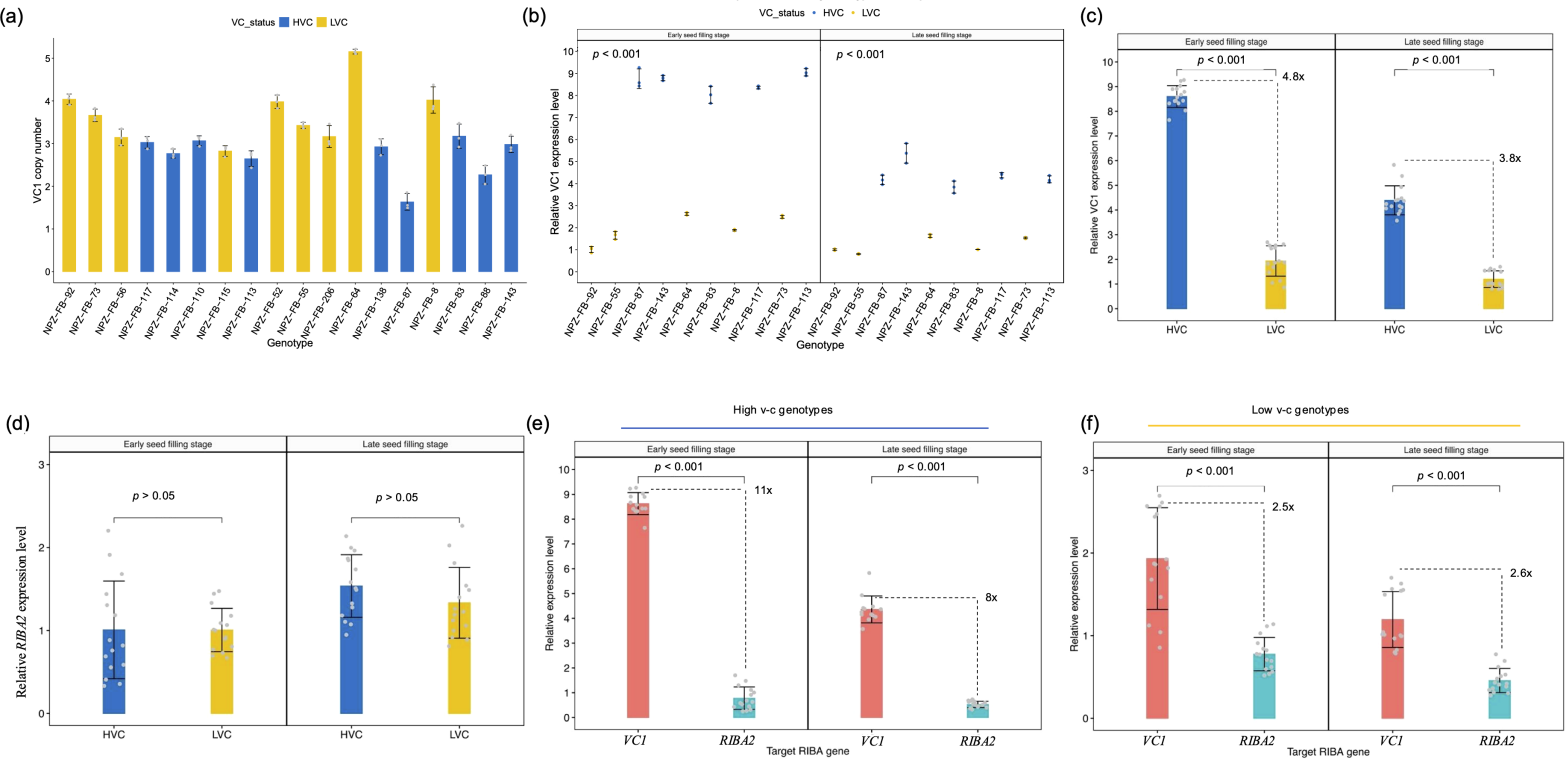
Copy number variation and transcription activities of RIBA genes. **(a)** *VC1* copy numbers across 18 faba bean genotypes, comprising high (HVC) and low vicine-convicine (LVC) lines (means ± SD, n = 3). **(b)** *VC1* relative expression levels during seed development (means ± SD, n = 3). **(c)** Comparisons of the relative expression levels between high and low v-c genotypes at early and late seed filling stages (means ± SD, n = 15). **(d)** Comparisons of relative levels of *RIBA2* transcripts between high and low v-c (means ± SD, n = 15). **(e)** Comparisons of *VC1* and *RIBA2* expression levels within high v-c genotypes (means ± SD, n = 15). **(f)** Comparison of *VC1* and *RIBA2* expression levels within low v-c genotypes (means ± SD, n = 15). Significance for comparisons between groups were determined using t-test.

The presence of multiple copies of *VC1* in faba bean reference genomes suggests a potential variable copy number for the gene. As a result, we determined *VC1* copy number through relative quantification by qPCR. The result confirmed that *VC1* shows copy number variations, with gene copies ranging from 2 to 5 across 18 faba bean genotypes (**Figure 4a**). Unexpectedly, low v-c genotypes tended to carry higher copy numbers than high v-c genotypes. This observation could mean that some of the gene variants are not functional or not expressed as was observed for the faba bean hilum color locus (Jayakodi et al., 2023). Therefore, we assessed the transcription activity of *VC1* and *RIBA2* genes by quantifying their relative expression levels using RT-qPCR in a subset of ten faba bean cultivars with varying v-c contents. These cultivars, selected to reflect the diversity in seed v-c levels, included genotypes representing all known *VC1* haplotypes. Furthermore, our focus was on whole seeds at the early stage of seed development, in which *VC1* expression is known to be the highest (Björnsdotter et al., 2021).

Expression analysis revealed significantly (P < 0.001) higher *VC1* expression levels in high v-c genotypes at early and late seed filling stages compared to low v-c genotypes (**Figure 4b**). High v-c genotypes showed about 5-fold and 4-fold more expression (P < 0.001) than low v-c genotypes at ESF and LSF, respectively (**Figure 4c**). In contrast to *VC1* differential expression, *RIBA2* displayed relatively consistent expression levels between high and low v-c genotype groups at both stages (**Figure 4d**). However, *VC1* expression was significantly higher than *RIBA2* expression. Within high v-c genotypes, *VC1* exhibited up to 11-fold more expression than *RIBA2* (**Figure 4e**), compared to 2.6-fold within low v-c genotypes (**Figure 4f**).

Importantly, despite fewer copy numbers in high v-c genotypes, the elevated expression of *VC1* suggests that it may not be sensitive to dosage. To validate this, we conducted a correlation analysis between *VC1* gene copy number and gene expression. Subsequently, we observed a negative correlation (**Supplementary Figure S2A**), indicating that higher copy number correlated with lower expression level. This negative correlation seems to be attributed to a shared genetic origin among low v-c genotypes, rather than interactive effects of variants resulting from antisense regulation. To verify this observation, we used a SNP genotyping dataset to trace the lineage of our low v-c lines across a broader panel of 347 faba bean genotypes. These genotypes were genotyped using the faba bean 50k Affymetrix chip. After filtering for missing data, minimum allele frequency, and heterozygosity, 13k polymorphic SNPs remained and were used for population structure analysis. The principal component analysis plot depicted a close clustering of the low v-c lines (**Supplementary Figure S2B**). This clustering pattern confirms a common genetic origin among low v-c genotypes, elucidating the observed negative correlation between *VC1* gene copy number and expression.

However, this observed correlation between high copy number and low expression suggests that not all *VC1* copies are expressed, potentially due to some regulatory mechanisms. To identify the functional variants, we sequenced cDNA fragments of *VC1*. As *VC1* variants can be differentiated by the mutations in exon 3 and 6, and SNPs within exon 4 and 5, we designed primers flanking this region and subsequently sequenced amplicons from four low v-c and five high v-c genotypes.

**Figure 5:**
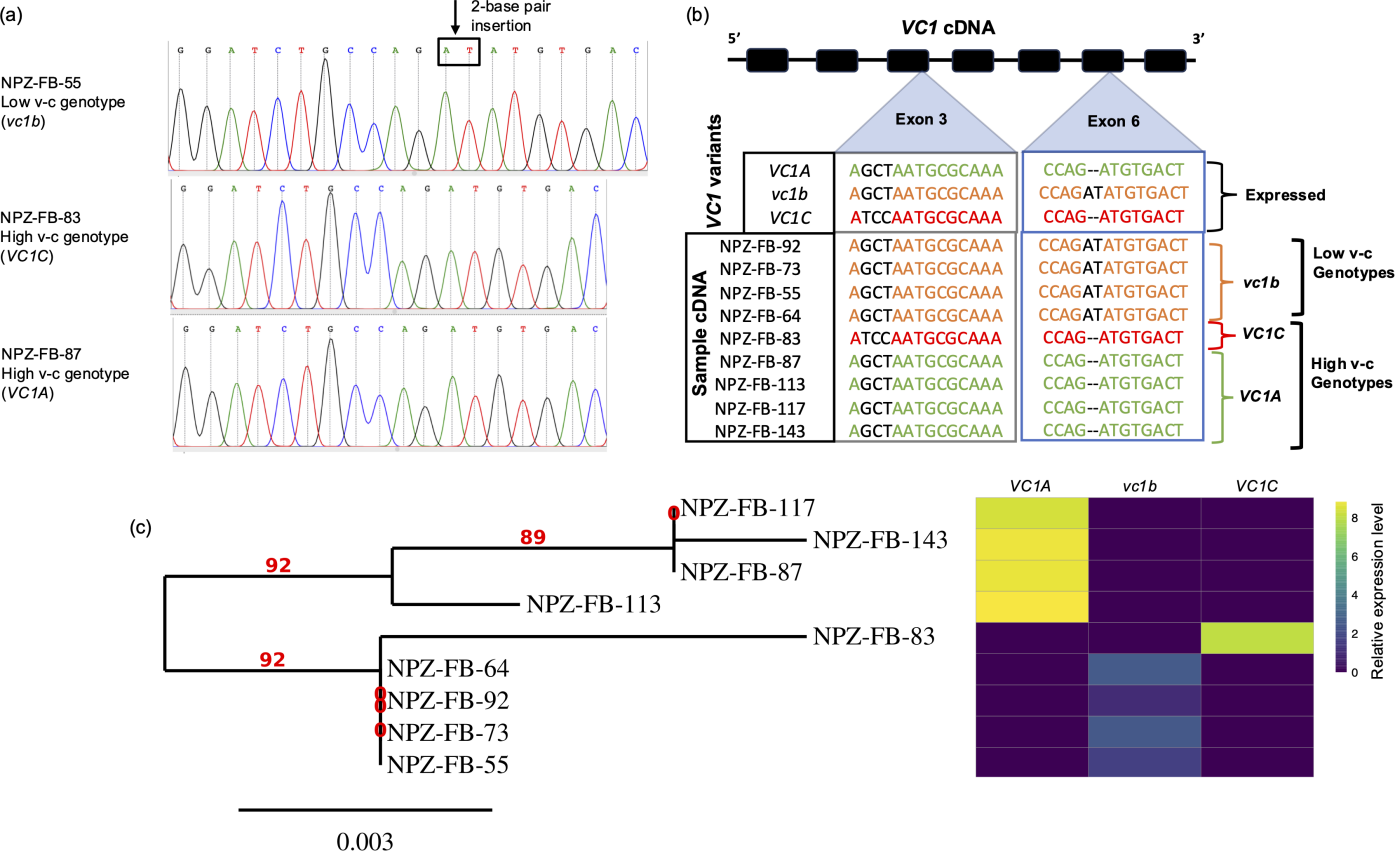
Identification of functional *VC1* variants. **(a)** Chromatograms obtained by Sanger sequencing of PCR fragments of VC1 cDNA show single peaks corresponding to only one variant of the gene. The position of the 2-base pair insertion is indicated by the arrow, and the nucleotides are enclosed in a box. **(b)** *VC1* cDNA analysis showing the expressed *VC1* variants. For each genotype, cDNA samples from two developmental stages were sequenced. In addition to the mutations shown here, single nucleotide polymorphisms in exons 4 and 5 were used to distinguish the variants (see **Supplementary Table S6**). **(c)** Phylogenetic tree showing the genetic relationships among the faba bean lines using maximum likelihood method, alongside a heatmap illustrating expression profiles for *VC1* variants.

The analysis of cDNA sequences showed that three *VC1* gene variants were expressed across genotypes, namely, high v-c genotypes can express *VC1A* or *VC1C*, while *vc1b* was detected in low v-c genotypes (**Figures 5a, b**; **Supplementary Table S6**). Surprisingly, only one gene variant was expressed per genotype, regardless of the total number of variants present. For instance, low v-c genotype NPZ-FB-92 carried four *VC1* gene copies, two each of *VC1B* and *VC1C*, but only *vc1b* mutant was expressed, as in all other low v-c genotypes. Similarly, high v-c genotype NPZ-FB-143 carried multiple gene copies including all three variants but expressed only *VC1A*. This demonstrates and explains why *VC1* was found dosage insensitive.

Additionally, we constructed a phylogenetic tree using *VC1* cDNA sequences for the sequenced subset. The genotypes clustered into two major groups (**Figure 5c**). The first group comprised only high v-c genotypes expressing *VC1A*, while the second group comprised two subgroups: low v-c genotypes expressing *vc1b* and high v-c genotypes expressing *VC1C*. This distinction within the second subgroup was primarily attributed to the presence or absence of the 2 bp insertion, suggesting it as a functional polymorphism within *VC1* capable of distinguishing genotypes based on v-c contents. We additionally sequenced *RIBA2* cDNA which overlaps with this *VC1* region, particularly within the GCHII domain. *RIBA2* cDNA sequences from both low v-c and high v-c genotypes did not carry any inactivating insertion (**Supplementary Table S6**).

### 3.3 *RIBA2* gene is a candidate v-c locus with localized SNPs that segregate with v-c phenotypes

**Figure 6:**
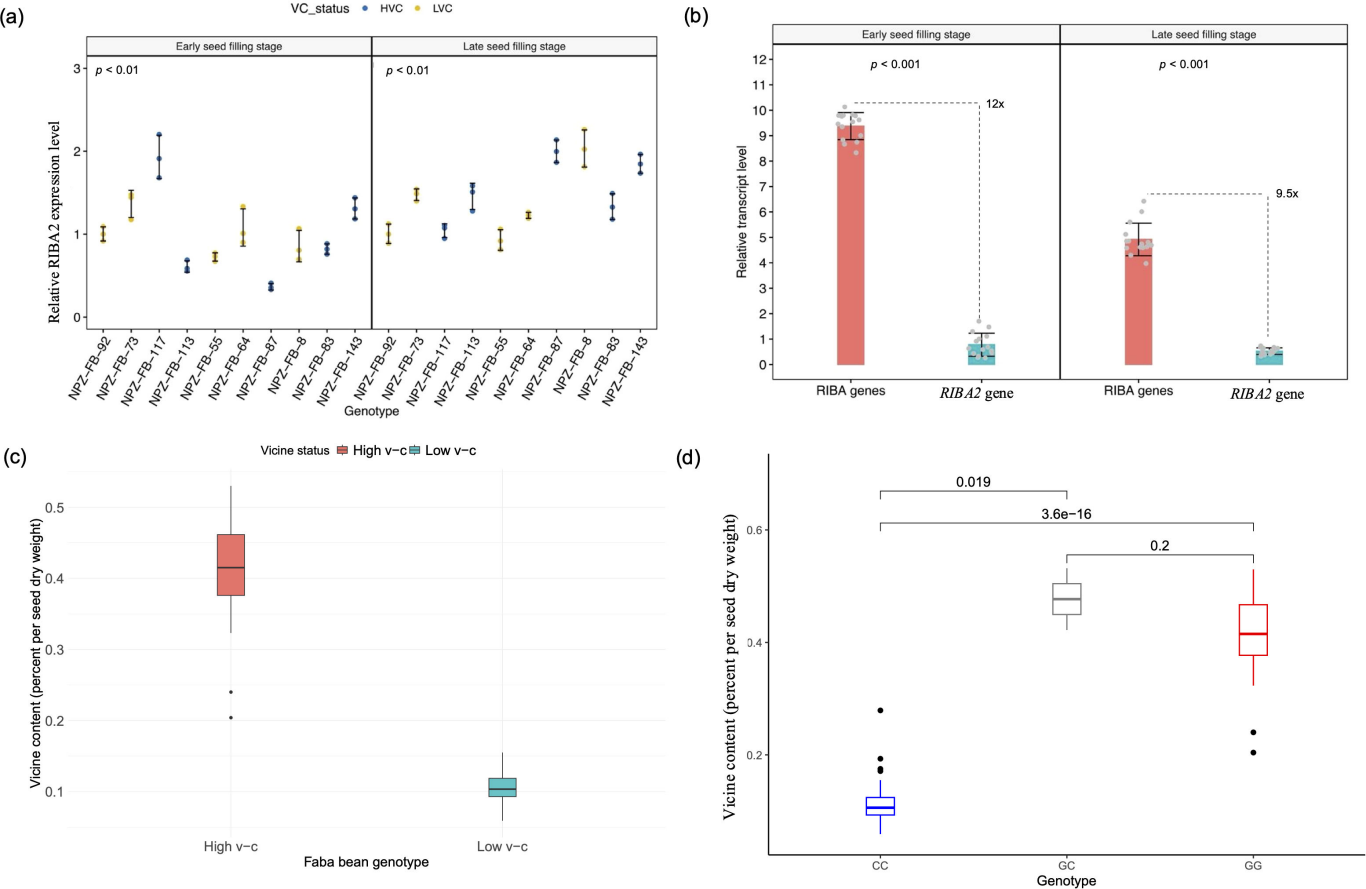
Analysis of *RIBA2* activity on vicine and convicine biosynthesis. **(a)** Relative expression levels of *RIBA2* across faba bean lines (means ± SD, n = 3). **(b)** Proportion of *RIBA2* expression relative to the combined RIBA gene (*VC1* and *RIBA2*) expression in high v-c lines at early and late seed filling stages (means ± SD, n = 15). Significance for comparisons between groups were determined using t-test. **(c)** Phenotypic differences in vicine content between high and low v-c faba bean genotypes **(d)** Allelic effects of a single nucleotide polymorphism within exon 6 of *RIBA2* gene in a panel of 97 diverse faba bean genotypes.

It is evident that *VC1* is not the only locus controlling v-c biosynthesis in faba bean. We identified a diverging homolog of *VC1* which may also be involved in v-c regulation. As earlier noted, analysis of protein sequences of this homologous gene revealed two functional domains, RibA and RibB, similar to the functional *VC1* protein domains, which are conserved across related legumes like chickpea, lupin, and pea. This high similarity and conservation of key amino acid residues among these proteins indicates that their roles as RIBA proteins are well conserved (see **Figure 2a**), suggesting that *RIBA2* is another functional RIBA locus in faba bean. Notable differential expressions were evident among individual faba bean lines for this gene, with observed variations tending to correlate with differences in v-c contents (**Figure 6a**).

Furthermore, when comparing the proportion of *RIBA2* expression relative to the combined expression of both RIBA genes in high v-c cultivars, we observed an average transcript level at least 9.5-fold lower, ranging up to 20-fold (**Figure 6b**). This would represent about 5-10% of v-c content relative to high v-c cultivars and corresponds to the observed phenotypic differences between low and high v-c cultivars (**Figure 6c**). These results suggest that this neighboring homolog of *VC1* is a candidate locus with minor effect in v-c regulation.

Additionally, two neighboring single nucleotide polymorphisms within exon 6 of the *RIBA2* gene segregate with v-c phenotypes. We evaluated the segregation pattern of a SNP in exon 6 of this gene in a diversity panel of 97 genotypes. The results showed that this polymorphism can differentiate low v-c from high v-c genotypes, where allele C segregated with low v-c phenotype while allele G segregated with high v-c phenotype (**Figure 6d**; **Supplementary Table S5**), demonstrating that the SNPs are tightly linked to v-c phenotypes and can be utilized for marker-assisted selection in v-c breeding.

### 3.4 Genetic variations in v-c contents involve polymorphisms associated with differential expression of *VC1* and *RIBA2* genes

It is commonly observed that high v-c faba beans exhibit substantial variations in v-c content, whereas low v-c faba beans show much narrower variation, significantly below this threshold (Khamassi et al., 2013; Puspitasari et al., 2022). This pattern suggests that the 2 bp inactivating insertion in *VC1*, although critical, may not fully explain the observed phenotypic diversity. Based on our findings, we propose that polymorphisms influencing the expression levels of *VC1* and *RIBA2*, in addition to the 2 bp insertion, contribute to the variations in v-c content (**Supplementary Figure S3**). Specifically, *VC1* expression levels can vary up to five-fold among faba bean genotypes, potentially accounting for much of the variability within high v-c group. In contrast, the 2 bp mutation in *VC1* appears to be the primary determinant of the sharp phenotypic difference between high and low v-c genotypes. Although *RIBA2* generally exhibits low expression and may play a minor role, it could be the sole contributor to v-c biosynthesis in low v-c lines lacking functional *VC1*. This may explain the narrow v-c variation observed in these genotypes, which remains substantially lower than in high v-c lines.

### 3.5 Implications of multiple *VC1* gene variants in molecular breeding for low vicine-convicine faba bean

It was observed that three different variants of *VC1* can be expressed. High v-c genotypes can express either *VC1A* or *VC1C*, while low v-c genotypes consistently express *vc1b*. Notably, *VC1C* shared close similarity with *vc1b*, having similar alleles at most SNP positions. This similarity caused genotypes expressing these variants to cluster under a major branch as observed in **Figure 5c**. Consequently, the existence of multiple *VC1* variants may present a significant challenge for marker-assisted selection of v-c content in faba bean breeding. Issues such as false heterozygous calls or incomplete segregation may arise due to the presence of multiple gene variants. To investigate this, we conducted genotyping assays using well-characterized low and high v-c genotypes. These assays involved single nucleotide polymorphic markers located within *VC1* gene, including some previously developed for v-c breeding. Since genomic DNA (gDNA) contains all gene variants, while complementary DNA (cDNA) only contains the expressed variant, we performed KASP genotyping assays with gDNA and cDNA as templates.

**Figure 7:**
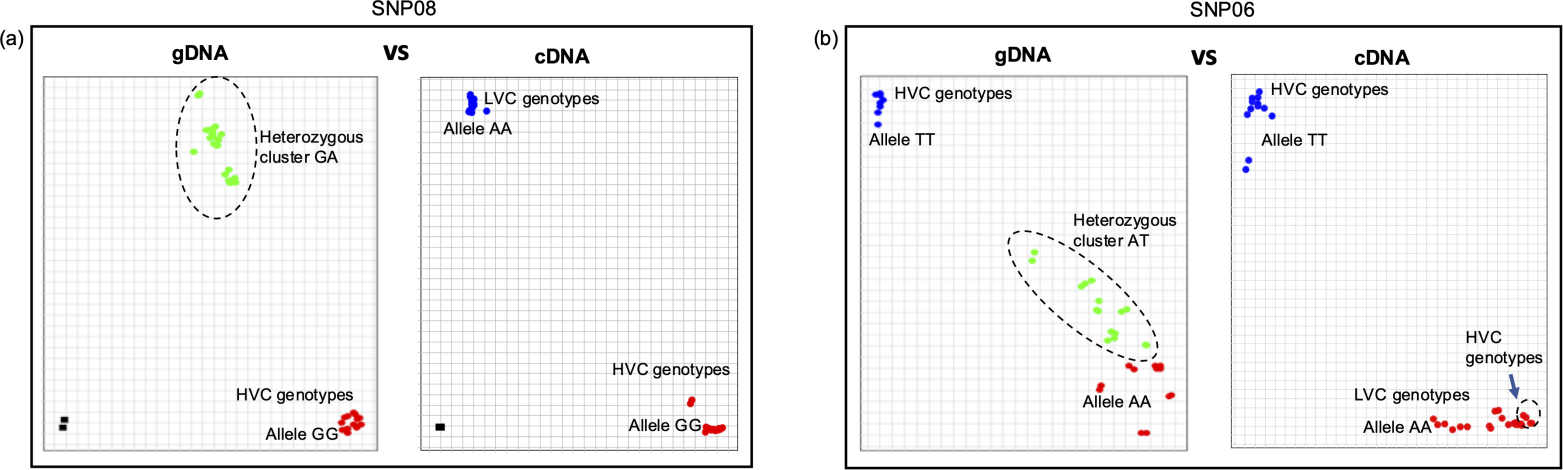
Multiple *VC1* copies can cause bias during marker analysis. Allelic discrimination plots for **(a)** 2 bp insertion in exon 6 and **(b)** an SNP within exon 5 of *VC1*. In both cases, the plots exhibited false heterozygous calls for all or most low vicine-convicine (v-c) (LVC) genotypes and some high v-c (HVC) genotypes when gDNA was employed as the template. In contrast, clear homozygous calls were observed with cDNA. Additionally, some high v-c genotypes expressing *VC1C* variant can cluster with low v-c genotypes, as observed in b (see arrow).

Our results revealed two key findings. Firstly, the presence of multiple variants could lead to bias in allele calls (**Figures 7a, b**; **Table 1**). In most cases, low v-c genotypes consistently exhibited false heterozygous signals when gDNA was used as a template. A similar trend can also occur in a few high v-c genotypes. However, when cDNA was used as the template, these genotypes were correctly identified as homozygous individuals. Likewise, in the assay targeting the 2 bp insertion, all low v-c genotypes were initially called as heterozygous when gDNA was used as the template, due to the presence of multiple copies, including both wild-type and mutant variants. However, these genotypes were correctly called as homozygous when cDNA was used as the template. Additionally, this observation further confirms that only one copy of *VC1* is expressed in each genotype analyzed.

**Table 1:** Comparison of allele calls between gDNA and cDNA for SNPs detected by KASP Assays showed that multiple copies of *VC1* often lead to inaccurate allele calls.

Genotype	SNP08		SNP05		SNP06	
	gDNA	cDNA	gDNA	cDNA	gDNA	cDNA
NPZ-FB-117	GG	GG	CC	CC	TT	TT
NPZ-FB-113	GG	GG	CC	CC	TT	TT
NPZ-FB-87	GG	GG	CC	CC	TT	TT
NPZ-FB-83	GG	GG	AA	AA	AT	AA
NPZ-FB-143	GA	GG	AC	CC	AT	TT
NPZ-FB-92	GA	AA	AA	AA	AA	AA
NPZ-FB-73	GA	AA	AC	AA	AT	AA
NPZ-FB-55	GA	AA	AA	AA	AA	AA
NPZ-FB-64	GA	AA	AA	AA	AA	AA
NPZ-FB-8	GA	AA	AA	AA	AA	AA

Allele calls were also confirmed using cDNA sequences (see **Supplementary Table S5**).

High and low v-c genotypes are highlighted in red and yellow, respectively.

Secondly, we observed that SNP alleles do not always segregate completely with the v-c phenotype. Low v-c alleles often segregate with some high v-c genotypes expressing *VC1C* variant. This results in the false clustering of these high v-c genotypes with low v-c genotypes, leading to inaccurate predictions of the v-c phenotype. (**Figure 7b**). This pattern was consistent with other *VC1*-based SNPs, except for the 2 bp insertion, which can efficiently distinguish the cultivars based on v-c contents. Beside this polymorphism, other SNPs within *VC1* were ineffective in predicting v-c contents in a diverse genetic background.

## 4 Discussion

### 4.1 *VC1* is multiallelic but exhibits single-copy expression

Until recently, the elucidation of genetic mechanisms underlying important traits in faba bean has been hindered by the unavailability of genomic tools for this crop, which has an enormous genome in which the largest chromosome is bigger than the entire human genome. This constraint equally impeded early endeavors to identify genes responsible for v-c biosynthesis in faba bean. In the absence of reference genomes, previous investigations relied on molecular markers generated by mapping mRNA contigs from faba bean to the genomes of closely related crops, such as *Medicago truncatula*, to identify regions controlling v-c content (Khazaei et al., 2015; Tacke et al., 2022). However, the involvement of *VC1* gene in v-c biosynthesis in faba bean was established recently and a frameshift mutation in this gene results in a loss-of-function, leading to a low v-c phenotype (Björnsdotter et al., 2021).

The availability of recently assembled *V. faba* reference genomes with functional gene annotations (Jayakodi et al., 2023) enabled us to map RIBA1 protein to these assemblies and identify all relevant RIBA genes associated with v-c biosynthesis. Our study revealed multiple *VC1* variants, primarily characterized by mutations in intron 3, exon 3 and 6. We found that *VC1* is affected by copy number variations, and that all low v-c genotypes carry both functional and non-functional variants, although only the non-functional variant is expressed. Previously, only two allelic forms of *VC1* were known, where low v-c faba beans carried only mutant *vc1*, while high v-c faba beans carried the wild-type variant (Björnsdotter et al., 2021). These wild-type and mutant alleles correspond to *VC1A* and *vc1b* variants, respectively. Our identification of an additional variant, *VC1C*, expressed by some high v-c lines, underscores the diversity of *VC1* genes and highlights the significance of utilizing multiple genotypes in our study. However, a major structural variation in intron 3 across the three *VC1* variants did not appear to have functional relevance as it did not associate with the expression pattern.

We observed a differential expression of VC1, consistent with previous reports between high and low v-c genotypes (Björnsdotter et al., 2021). Surprisingly, copy number did not correlate with phenotypic expression. Although we observed a negative correlation between copy number and gene expression, this was due to the influence of low v-c genotypes sharing a single genetic source. These genotypes exhibited high copy numbers but lower expression levels. This observation was supported by cDNA sequencing which showed that each genotype expressed only one *VC1* variant despite having multiple variants in the genome. Previous reports on the transformation of hairy roots of Hedin with an additional copy of functional *VC1* did not result in increased vicine accumulation (Björnsdotter et al., 2021). This confirms that despite copy number variation, *VC1* is not sensitive to dosage.

The mechanisms involved in *VC1* dosage compensation are unclear; however, certain mechanisms have been proposed for genes with multiple copies. These mechanisms could involve complex processes at different transcription stages (Vaquerizas et al., 2009; Woodwark and Bateman, 2011), including microRNAs (miRNAs) that can activate or repress the transcription of duplicated or copy number variable (CNV) genes (Vasudevan et al., 2007; Li et al., 2008; Bartel, 2009; Woodwark and Bateman, 2011; Chang and Liao, 2012), imprinted or monoallelically expressed genes (Pauler et al., 2007), and DNA methylation (Suzuki and Bird, 2008; Law and Jacobsen, 2010).

The regulation of *VC1* differential expression between low and high v-c genotypes and the impact of the 2 bp insertion carried by low v-c genotypes on expression levels remain unclear. Differences in the expression of *VC1* genes can also stem from variations within the regulatory elements. Elements like enhancers and silencers have the potential to amplify or suppress the gene expression of the target gene (Bulger and Groudine, 2011; Kolovos et al., 2012). Upstream and downstream of *VC1* are various variants, such as short tandem repeats, InDels and SNPs. Structural differences within this region could potentially affect one or more regulatory elements. The interaction among these diverse regulatory components, their interplay with target promoters, and the involvement of epigenetic modifications can intricately regulate the expression of this gene (Bulger and Groudine, 2011; Kolovos et al., 2012; Cremer and Cremer, 2001; Maeda and Karch, 2011; Yang and Corces, 2011).

### 4.2 *RIBA2* is a functional RIBA locus in faba bean and a candidate for a minor effect v-c locus

The variation in *VC1* expression alone does not correlate with observed phenotypic differences among faba bean genotypes. Subsequent analysis of *VC1* cDNA in this study aligns with findings reported by Björnsdotter et al. (2021), that all low v-c cultivars express *vc1b* variant carrying 2 bp insertion in exon 6 which results in a non-functional protein. This implies that *VC1* is not active in low v-c genotypes. However, as previously mentioned, this mutation only causes a significant reduction in v-c content but does not entirely eliminate it. For instance, the seed v-c content of the first low v-c genotype, initially reported by Duc et al. (1989) is approximately 0.04%, which is about 1/10 to 1/20 of high v-c contents. This substantial reduction in v-c content due to a single gene mutation highlights the major effect of *VC1* locus in v-c phenotype regulation and equally suggests the involvement of another gene with a minor effect.

In addition to the already known *VC1*, we identified a homologous gene, *RIBA2*, encoding putative bi-functional riboflavin proteins in faba bean genome. The distinct structure of the *RIBA2* gene compared to *VC1* suggests that it represents a second RIBA locus in faba bean. *RIBA2* is a bi-functional riboflavin gene with two catalytic domains, RibB and RibA, encoding DHBPS and GCHII enzymes, respectively. RIBA enzymes are known for their involvement in the riboflavin biosynthetic pathway in plants (Hiltunen et al., 2012). However, Björnsdotter et al. (2021) demonstrated that RIBA enzymes are also involved in v-c production in faba bean where v-c are synthesized in a three-step pathway that starts from the GTP cyclohydrolase II function of RIBA proteins. While *VC1* and *RIBA2* share nearly identical functional domains, *RIBA2* lacks the inactivating insertion present in *vc1b* mutant. Detailed analysis of *VC1* and *RIBA2* amino acid sequences showed conservation in all key amino acid residues necessary for catalytic activities, including those required for binding zinc ions essential for GCHII activity as demonstrated by Kaiser et al. (2002). These key amino acid residues are well conserved in *VC1* and *RIBA2* and other RIBA proteins within Fabaceae family but lacking in *vc1b*.

Subsequent analysis shows that *RIBA2* is expressed at much lower levels than *VC1* in seeds. Similarly, there was a significantly lower expression level relative to the combined riboflavin gene transcripts in high v-c genotypes, aligning with the observed phenotypic differences between low and high v-c genotypes. Previous studies consistently emphasize the *VC1* locus as a major determinant in v-c regulation (Ramsay et al., 1995; Gutierrez et al., 2006; Khazaei et al., 2015; Tacke et al., 2022). Our study substantiates this hypothesis, indicating several-fold higher expression of *VC1* relative to the homolog, *RIBA2*. These findings suggest that this homologous gene is a candidate for a minor effect locus for v-c content. The expression of this minor effect gene may explain why mutation in *vc1* does not eliminate v-c completely in affected genotypes. Tacke et al. (2022) used a transcript-based annotation method to identify a differentially expressed contig associated with v-c contents, as well as another, non-differentially expressed contig which mapped to the *VC1* locus. However, they could not fully decipher the locus due to the lack of genomic data.

It is essential to validate the involvement of *RIBA2* in v-c biosynthesis through mutant analysis, particularly in a *vc1* background. However, the lack of reliable transformation methods and mutant resources in faba bean significantly hinders functional gene validation through knockouts (O’Sullivan and Angra, 2016). Moreover, given that riboflavin biosynthesis is essential for fundamental cellular processes and overall plant development, it remains uncertain whether simultaneous loss-of-function mutations in both *VC1* and *RIBA2* would be viable.

### 4.3 The complexity of *VC1* locus limits the efficiency of localized SNPs for marker-assisted selection in v-c breeding

Various molecular markers have been developed to facilitate breeding of low v-c faba beans (Tacke et al., 2022; Khazaei et al., 2017). However, most existing v-c markers target polymorphisms within *VC1* genes. While these markers have proven valuable in some contexts, their efficiencies may be limited due to complex nature of *VC1*. Our research revealed that multiple *VC1* copies can lead to inaccurate allele calls, resulting in false clusters during marker analysis. This often happens because of the existence of closely homologous sequences in the genome (Makhoul et al., 2020). Consequently, conventional KASP assays may require optimization to improve accuracy (Makhoul et al., 2020). Nevertheless, the applicability of these SNPs might be confined to specific genetic backgrounds. In a diverse genetic background, comprising all expressed *VC1* variants, SNPs often do not segregate fully with the phenotype. Hence, these polymorphisms may not be efficient for MAS of v-c in faba bean breeding. Therefore, we strongly advise caution when utilizing *VC1*-based molecular markers for v-c content selection in breeding programs. Overreliance on only these markers for selection may inadvertently lead to incorrect prediction of v-c contents.

However, our study highlights the functional significance of the 2 bp insertion in exon 6 as a reliable polymorphism in *VC1* that effectively distinguishes genotypes based on the v-c phenotype. Optimizing the KASP assay targeting this polymorphism could significantly enhance its efficiency and specificity, particularly given the potential for inaccurate clustering due to multiple gene copies. Moreover, we have shown that SNPs within *RIBA2* show consistent segregation with v-c contents. These SNPs can accurately predict v-c content without bias and will be valuable for faba bean breeding.

## 5 Conclusion

We have demonstrated that *VC1* exists in multiple copies and shows CNV. However, copy number does not correlate with gene expression and suggests a tight regulation of the gene. Multiple *VC1* variants were expressed among low and high v-c genotypes which complicates molecular marker development for breeding. We also identified a diverging homolog of *VC1*, *RIBA2*, which shares nearly identical RIBA domains with *VC1*. Our results show that *RIBA2* has two functional domains similar to the active *VC1* genes, with highly conserved key amino acid residues, indicating well-conserved roles as RIBA proteins. Our findings suggest that this homologous gene is a candidate minor effect v-c locus, and its involvement can be validated using gene editing knockouts.

## Data Availability

The original contributions presented in the study are included in the article/**Supplementary Material**. Further inquiries can be directed to the corresponding author.
