# Supplementary material for: Multiple copy number variants of VC1 gene reveal single-copy expression as a key determinant of vicine content
Source: Front Plant Sci. 2025 Jun 13;16:1565210. doi: 10.3389/fpls.2025.1565210 (PMC12202586; doi:10.3389/fpls.2025.1565210)
Supplement: Supplementary file 1 [file DataSheet1.pdf]

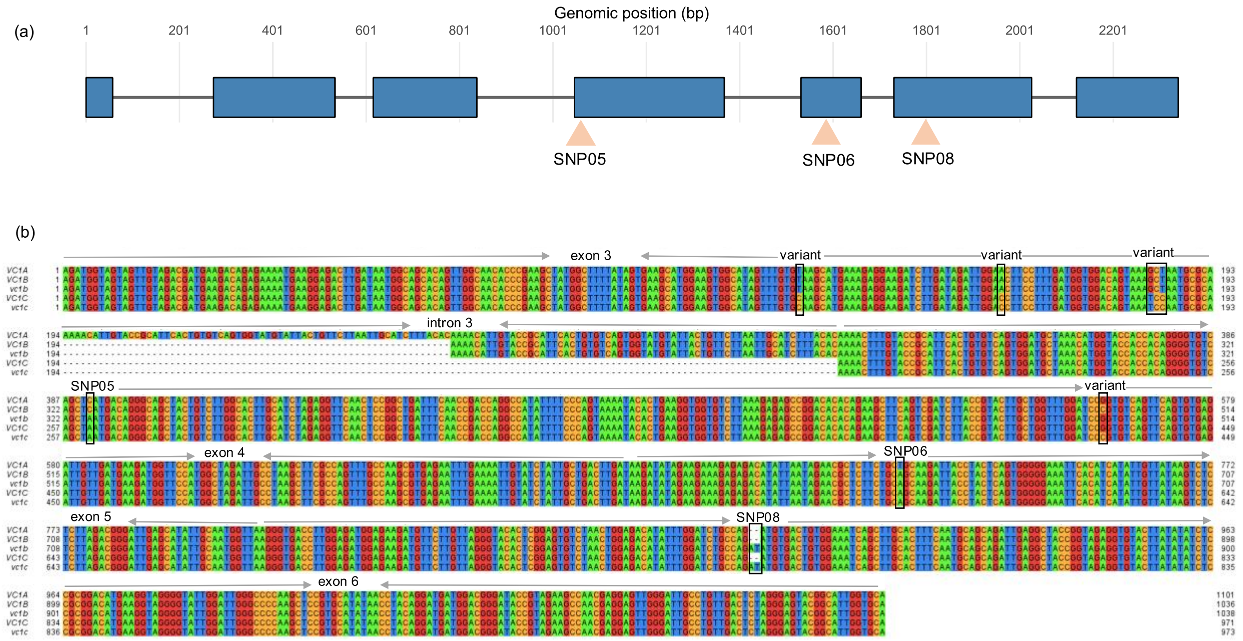

Supplemental Figure 1: Structural variations in *VC1* genes. (a) Schematic representation of the *VC1* gene from the Hedin reference genome, showing the positions of KASP primers. (b) Alignment of reference *VC1* genes from Hedin and Tiffany showing polymorphisms in the coding regions that can distinguish different gene variants.

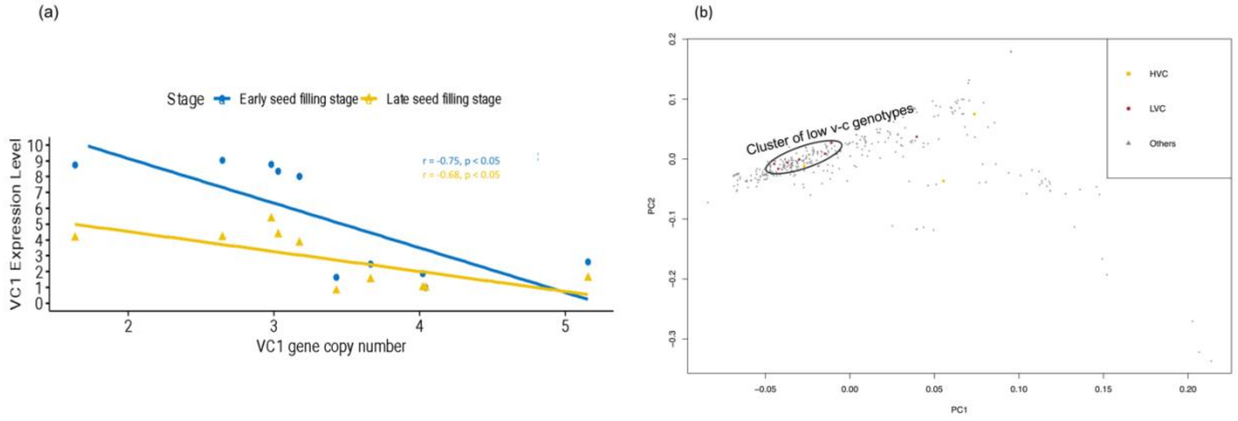

Supplemental Figure 2: **Tracing the source of low vicine and convicine (v-c) trait**. (a) Correlation analysis showing the relationship between *VC1* copy number and *VC1* expression (means ± SD, n = 3). (b) Principal component analysis showed that most low v-c genotypes cluster together as they share a single genetic origin, as shown in dark circle.

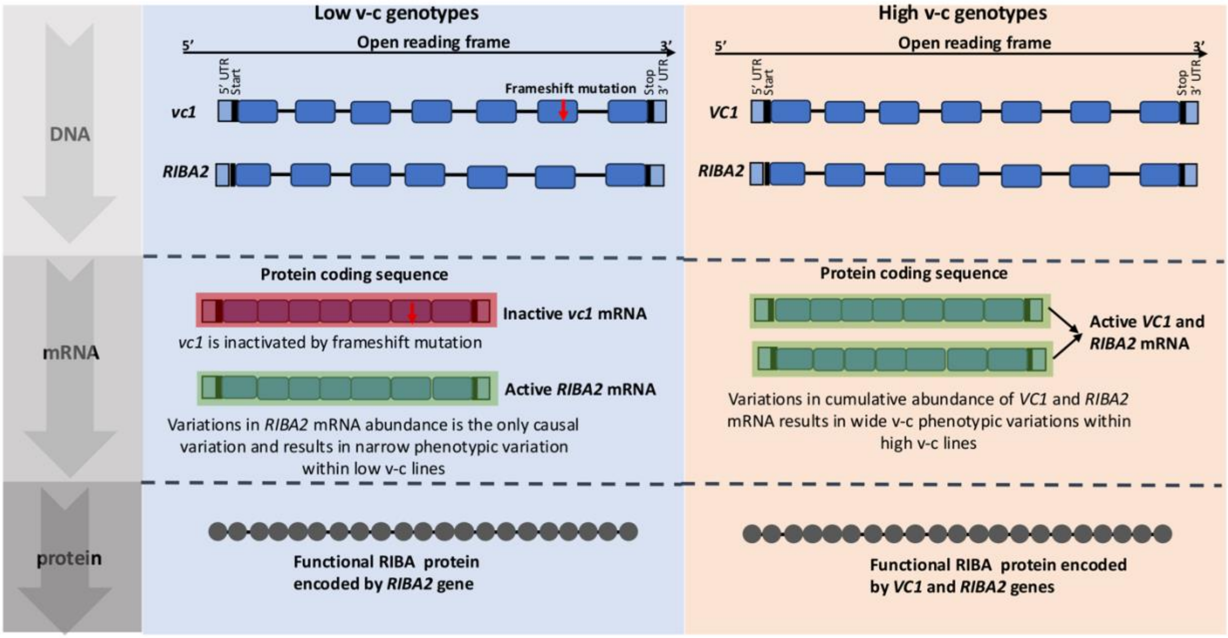

Supplemental Figure 3: Schematic representation of genetic variations in vicine and convicine (v-c) contents involving *VC1* and *RIBA2* genes.
